# Supplementary material for: Increased levels of mitochondrial import factor Mia40 prevent the aggregation of polyQ proteins in the cytosol
Source: EMBO J. 2021 Jun 30;40(16):e107913. doi: 10.15252/embj.2021107913 (PMC8365258; doi:10.15252/embj.2021107913)
Supplement: Supplementary file 2 — Expanded View Figures PDF [file EMBJ-40-e107913-s013.pdf]

Expanded View Figures

Figure EV1. Co-expression of Q97-GFP and *GAL*-Mia40 results in minor changes of mitochondrial morphology.

A Cells were grown to mid-log phase in glucose-containing medium, shifted to galactose-containing medium for 4 h, and analyzed by 3D fluorescence microscopy. Fluorescence micrographs are z stacks subjected to deconvolution. DIC, differential interference microscopy. Bar, 5 μm.

B Cells were grown to mid-log phase in medium containing glycerol (3%) and ethanol (2%) as carbon sources, shifted to galactose-containing medium for 4 h, and analyzed by 3D fluorescence microscopy. Fluorescence micrographs are maximum intensity projections of z stacks. Asterisks indicate representative cells exhibiting interconnected, "curly" mitochondria. Bar, 5 μm.

 © 2021 The Authors

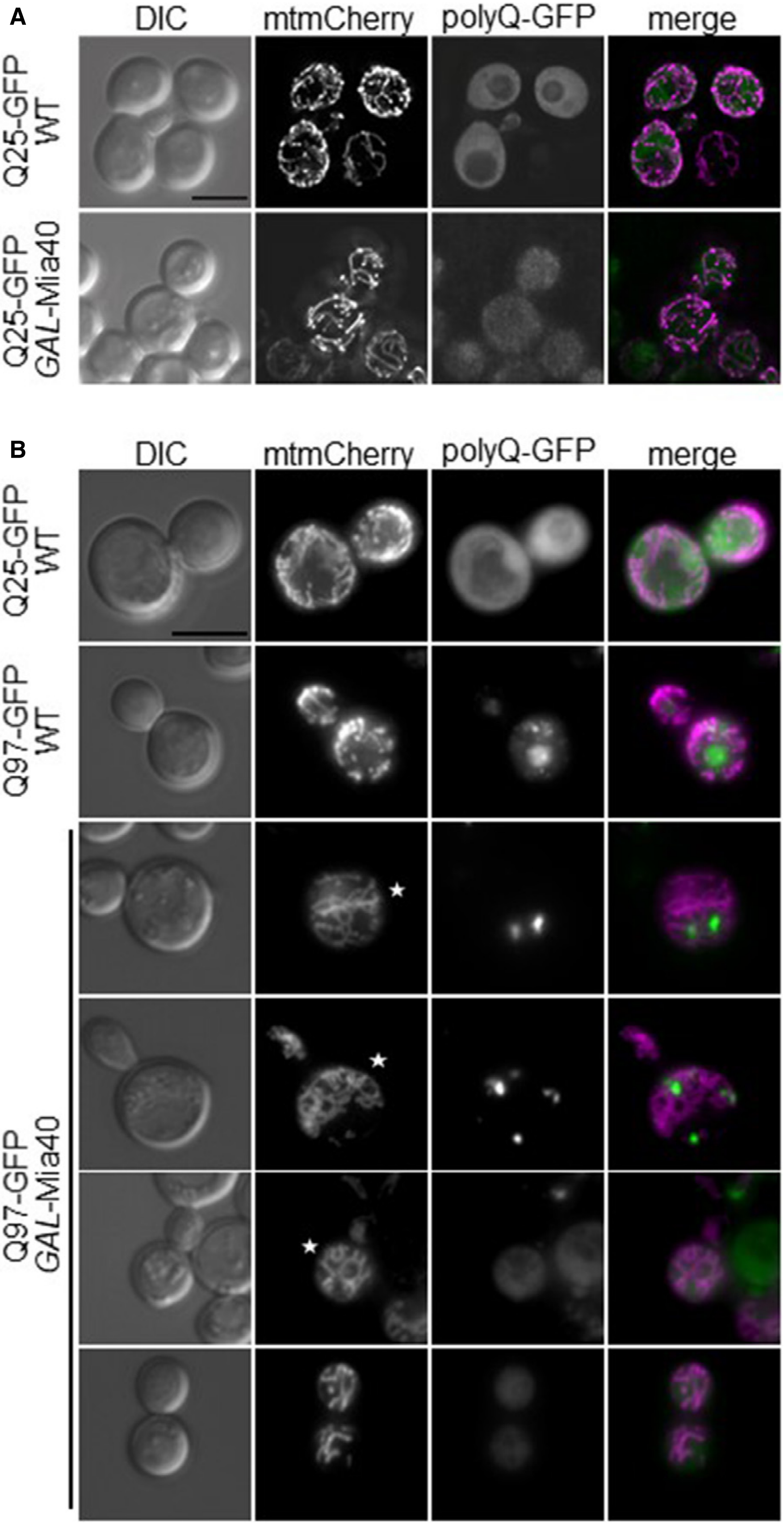
A
DIC
mtmCherry
polyQ-GFP
merge
Q25-GFP WT
Q25-GFP GAL-Mia40
B
DIC
mtmCherry
polyQ-GFP
merge
Q25-GFP WT
Q97-GFP WT
Q97-GFP GAL-Mia40

Figure EV1.

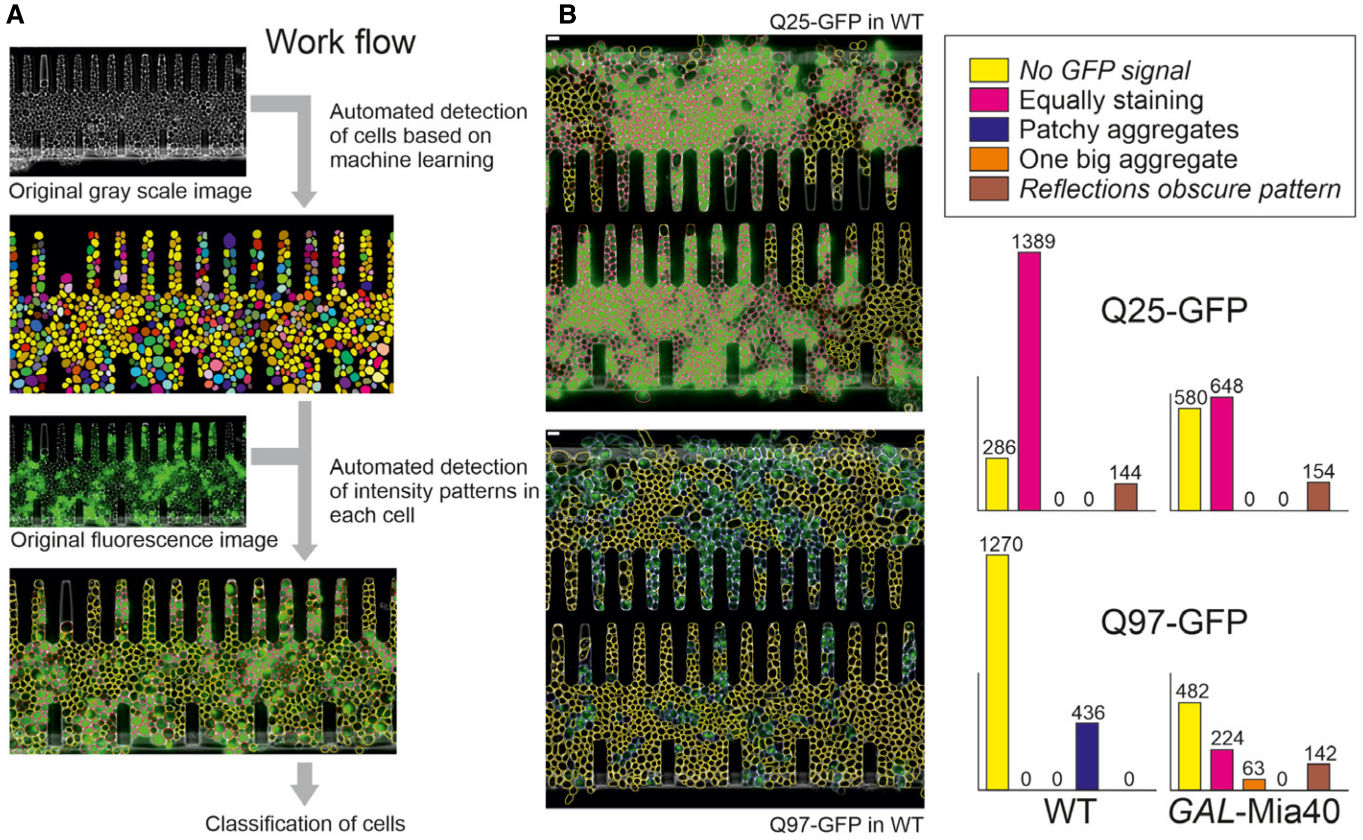

Figure EV2. The different patterns of aggregate formation can be quantified by an automated method trained with a machine learning approach.

A An automated detection algorithm was established that was trained by use of 154 original grayscale images of growing yeast cells for which each cell had been outlined by hand by visual inspection. Machine learning optimized cell detection before the patterns of fluorescence were again detected manually in a training set from which the algorithm learned to distinguish five different classes of patterns.

B Examples of these automatically detected classes of cells are shown in which the five categories are indicated by different colors. The number of cells in each category is shown here for different yeast strains as indicated. Bars, 4 µm.

Figure EV3. Expression of Q97-GFP does not block the import of the Mia40 substrate Cox19.

A Drop dilution experiment of the indicated strains.

B Cells of the indicated strains were grown in galactose medium overnight. Cells were lysed and either directly loaded to the gel (T, total) or separated into pellet (P) and supernatant (S) fractions by centrifugation. The indicated proteins were detected by Western blotting.

C Wild-type cells containing the Q97-GFP expression or empty plasmids were radiolabeled for 3 min with ^{35}S-methionine before labeling was quenched by addition of excess amounts of non-radioactive methionine. At the time points indicated, proteins were precipitated with trichloroacetic acid before the alkylating agent methyl-polyethylene glycol (24)-maleimide (mmPEG$_{24}$) was added which shifts the size by about 2 kDa per free reduced thiol group. For control, tris(2-carboxyethyl) phosphine (TCEP) was added to reduce all disulfide bonds.

D Wild-type cells carrying the *Δura3* marker allele were transformed with plasmids for the expression of Oxa1-Ura3, Δ5-Oxa1-Ura3 (lacking the N-terminal five residues), or ΔN-Oxa1-Ura3 (lacking the entire 42 residues of the mitochondrial targeting sequence of Oxa1). Serial dilutions were dropped on plates containing or lacking uracil.

E The three indicated proteins were expressed in wild-type cells. Please note that Q97-GFPnF expression does not lead to a detectable fluorescent signal. Cell boundaries are indicated by dashed lines.

Source data are available online for this figure.

 © 2021 The Authors

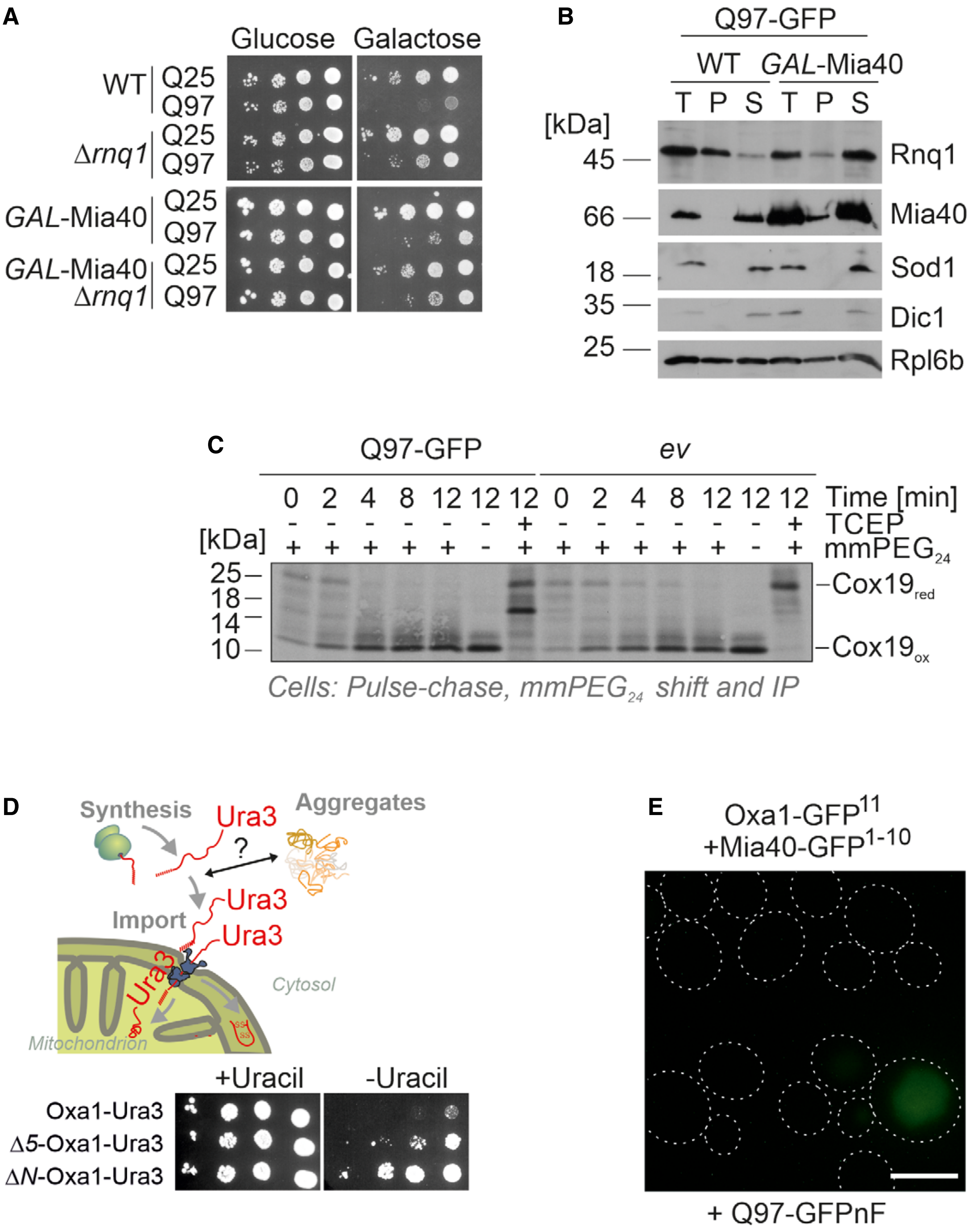
A
Glucose
Galactose
WT
Q25
Q97
Δrnq1
Q25
Q97
GAL-Mia40
Q25
Q97
GAL-Mia40
Δrnq1
Q25
Q97
B
Q97-GFP
WT
GAL-Mia40
T P S T P S
[kDa]
45
66
18
35
25
Rnq1
Mia40
Sod1
Dic1
Rpl6b
C
Q97-GFP
ev
0 2 4 8 12 12 12 0 2 4 8 12 12 12 Time [min]
TCEP
[kDa]
mmPEG24
25
18
14
10
Cox19red
Cox19ox
Cells: Pulse-chase, mmPEG24 shift and IP
D
Synthesis
Ura3
Aggregates
?
Ura3
Import
Ura3
Ura3
Cytosol
Mitochondrion
+Uracil
-Uracil
Oxa1-Ura3
Δ5-Oxa1-Ura3
ΔN-Oxa1-Ura3
E
Oxa1-GFP11
+Mia40-GFP1-10
+ Q97-GFPnF

Figure EV3.

Figure EV4. Overexpression of carrier proteins prevents cell growth.

A, B The respective yeast strains were analyzed by a drop dilution experiment and fluorescence microscopy. Please note that the absence of Hsp104 suppresses the formation of toxic polyQ aggregates. Cell boundaries are indicated by dashed lines. Bars, 5 µm.

C The indicated proteins were co-expressed with Q97-GFP. Lactate-grown precultures were dropped onto glucose and galactose plates. Please note that without pre-conditioning only individual cells were able to escape polyQ toxicity. Suppressors are indicated by red protein names (cf. Fig 7B). Such escaping cells were only observed if specific proteins were co-expressed with Q97-GFP.

D–F The indicated strains were grown to mid-log phase in lactate medium before serial dilutions were dropped on the indicated plates and incubated at 30°C. OM depicts expression of linker proteins with an N-terminal outer membrane anchor corresponding to the residues 1–36 of Tom70. Gal., galactose and *ev*, empty vector.

Source data are available online for this figure.

 © 2021 The Authors

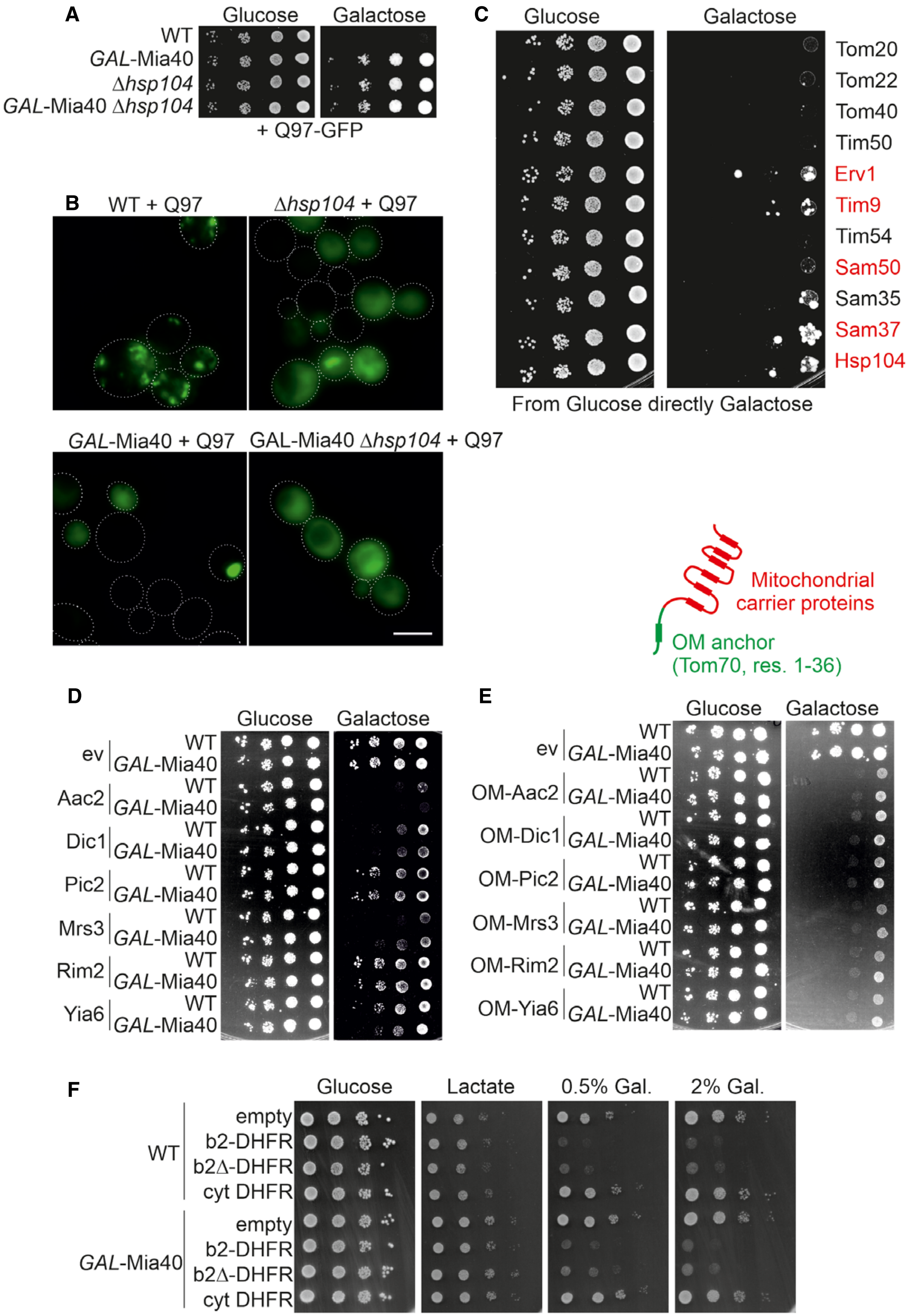
A
Glucose
Galactose
WT
GAL-Mia40
Δhsp104
GAL-Mia40 Δhsp104
+ Q97-GFP
B
WT + Q97
Δhsp104 + Q97
GAL-Mia40 + Q97
GAL-Mia40 Δhsp104 + Q97
C
Glucose
Galactose
Tom20
Tom22
Tom40
Tim50
Erv1
Tim9
Tim54
Sam50
Sam35
Sam37
Hsp104
From Glucose directly Galactose
Mitochondrial carrier proteins
OM anchor (Tom70, res. 1-36)
D
Glucose
Galactose
ev
Aac2
Dic1
Pic2
Mrs3
Rim2
Yia6
WT
GAL-Mia40
E
Glucose
Galactose
ev
OM-Aac2
OM-Dic1
OM-Pic2
OM-Mrs3
OM-Rim2
OM-Yia6
WT
GAL-Mia40
F
Glucose
Lactate
0.5% Gal.
2% Gal.
WT
GAL-Mia40
empty
b2-DHFR
b2Δ-DHFR
cyt DHFR

Figure EV4.

Figure EV5. Overexpression of Mia40 does not lead to a generally increased stress resistance.

A Cells were grown on lactate medium. Tenfold dilutions were dropped on the respective plates and incubated at 30°C (A) or 37°C (B). The concentrations of diamide or paraquat were 0.5 and 1 mM, respectively; *ev*, empty vector.

B–D The expression of MIA40, ΔN-MIA40, or Q25-GFP does not affect mitochondrial morphology. SH-SY5Y cells were analyzed by immunocytochemistry as indicated. TOM20 was used as mitochondrial marker. Whereas MIA40 co-localizes with TOM20, ΔN-MIA40 is not efficiently targeted into mitochondria. Bars, 20 μm.

Source data are available online for this figure.

© 2021 The Authors

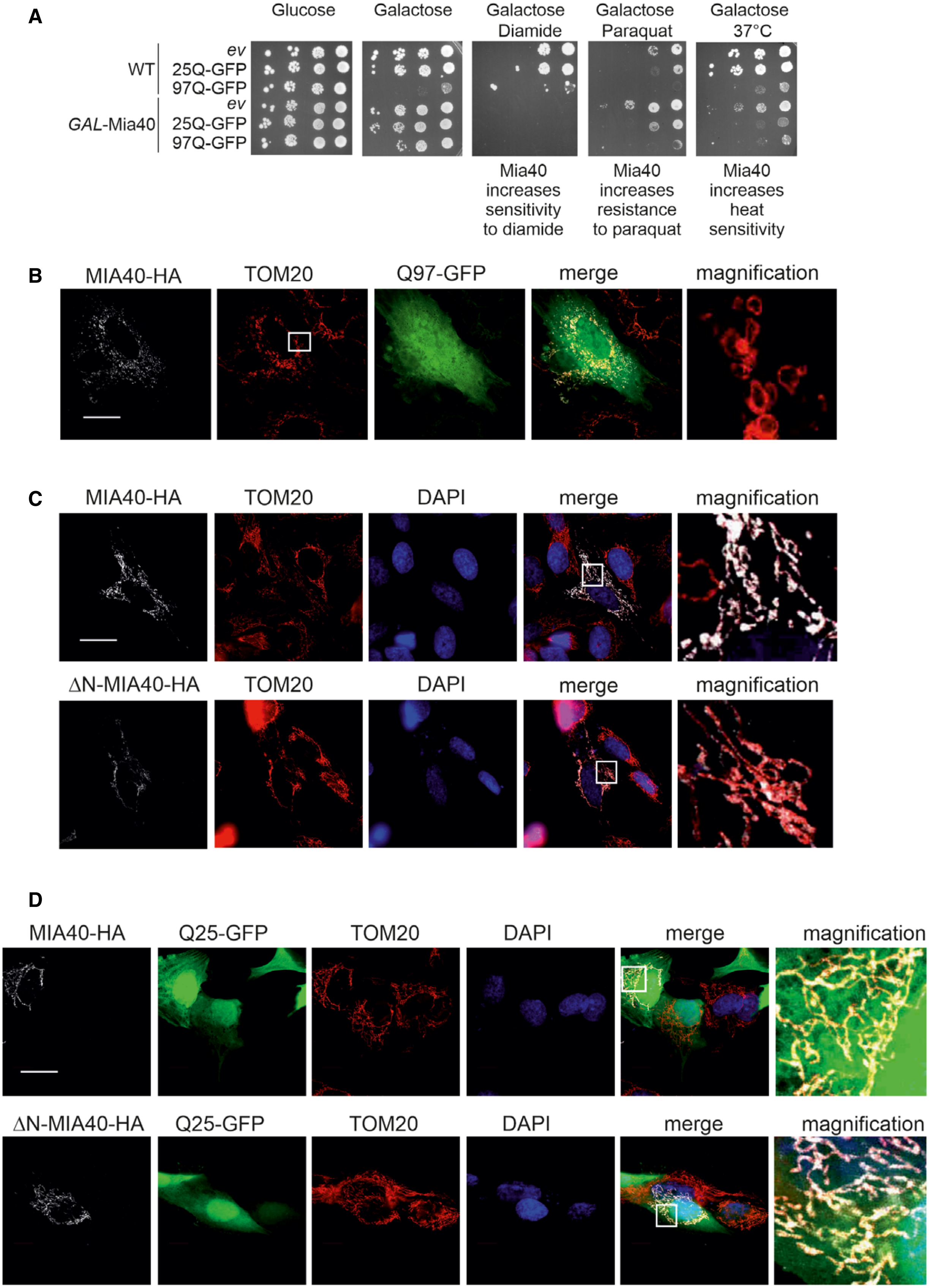
A
Glucose
Galactose
Galactose Diamide
Galactose Paraquat
Galactose 37°C
WT
ev
25Q-GFP
97Q-GFP
GAL-Mia40
ev
25Q-GFP
97Q-GFP
Mia40 increases sensitivity to diamide
Mia40 increases resistance to paraquat
Mia40 increases heat sensitivity
B
MIA40-HA
TOM20
Q97-GFP
merge
magnification
C
MIA40-HA
TOM20
DAPI
merge
magnification
ΔN-MIA40-HA
TOM20
DAPI
merge
magnification
D
MIA40-HA
Q25-GFP
TOM20
DAPI
merge
magnification
ΔN-MIA40-HA
Q25-GFP
TOM20
DAPI
merge
magnification

Figure EV5.
