# Supplementary material for: Increased levels of mitochondrial import factor Mia40 prevent the aggregation of polyQ proteins in the cytosol
Source: EMBO J. 2021 Jun 30;40(16):e107913. doi: 10.15252/embj.2021107913 (PMC8365258; doi:10.15252/embj.2021107913)
Supplement: Supplementary file 9 — Movie EV4 [file EMBJ-40-e107913-s001.zip › Movie_EV4.docx]

**Movie EV4. Expression of Q97-GFP in *GAL*-Mia40 cells.**

The growth of yeast cells was visualized using microfluidics. Cells were grown on chips with constant perfusion of glucose selective medium (SD-Leu-Ura) until most cavities were filled with cells (typically overnight). Then induction was started by changing the medium to SC+2%Galactose-Leu-Ura. At the same time, time-lapse acquisition was initiated. Cells were imaged using an inverted Nikon Ti-E microscope. Fluorescence illumination was achieved using LED light (Lumencor) and emitted light was collected using a 60x N.A. 1.4 objective and a CMOS camera Hamamatsu Orca Flash 4.0. An automated stage was used to follow up to 34 different fields of view in parallel over the course of the experiment. Images were acquired every 10 min for a total duration of 48 h using NIS software.
