# Supplementary figures and images for: Increased levels of mitochondrial import factor Mia40 prevent the aggregation of polyQ proteins in the cytosol
Source: EMBO J. 2021 Jun 30;40(16):e107913. doi: 10.15252/embj.2021107913 (PMC8365258; doi:10.15252/embj.2021107913)

Fig.EV3A

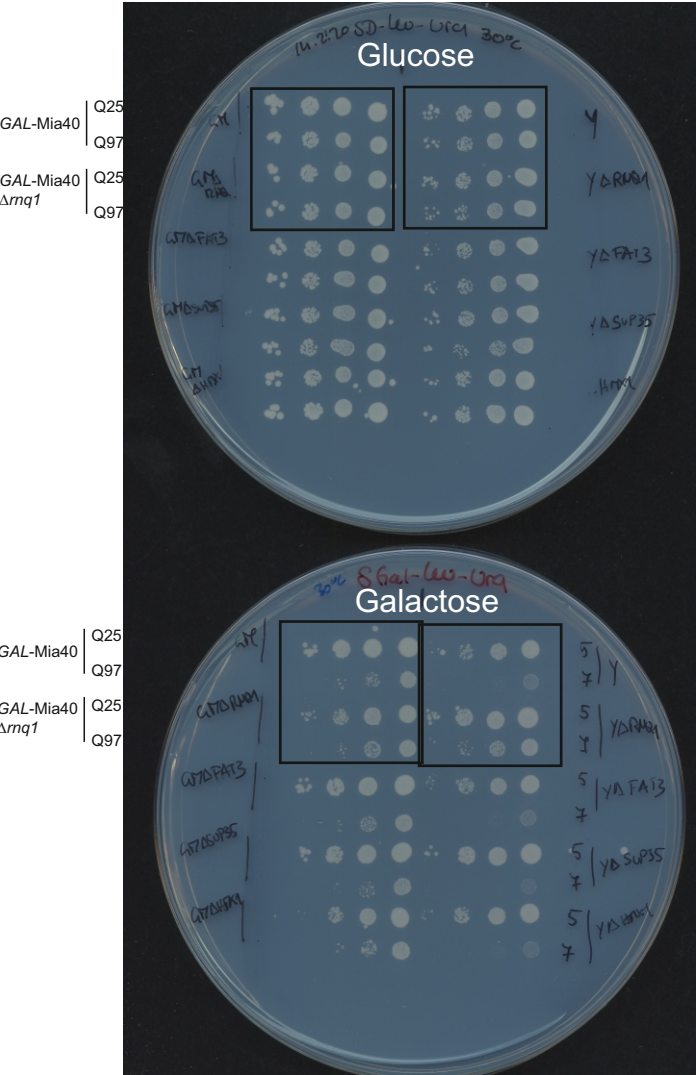

Fig.EV3B

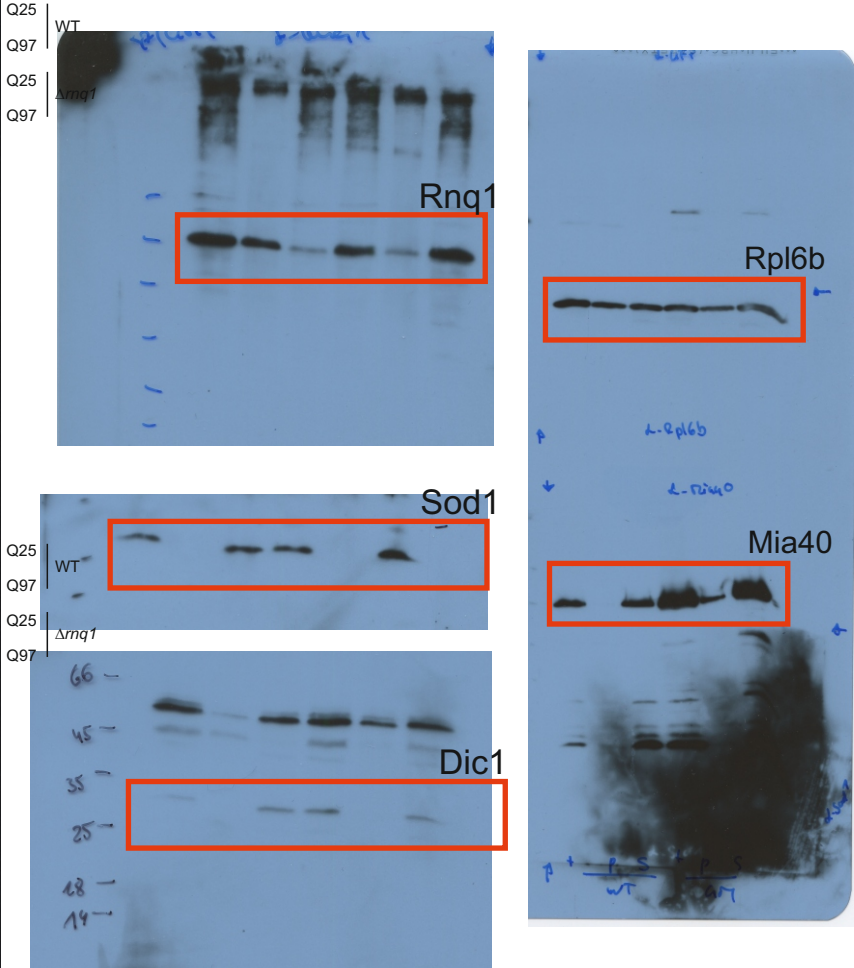

Fig.EV3C

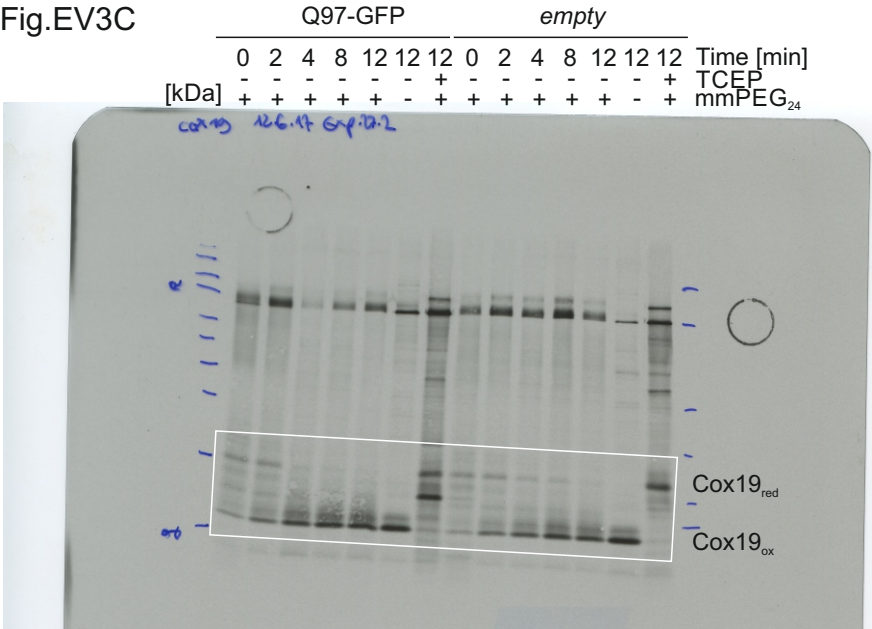

Fig.EV3D

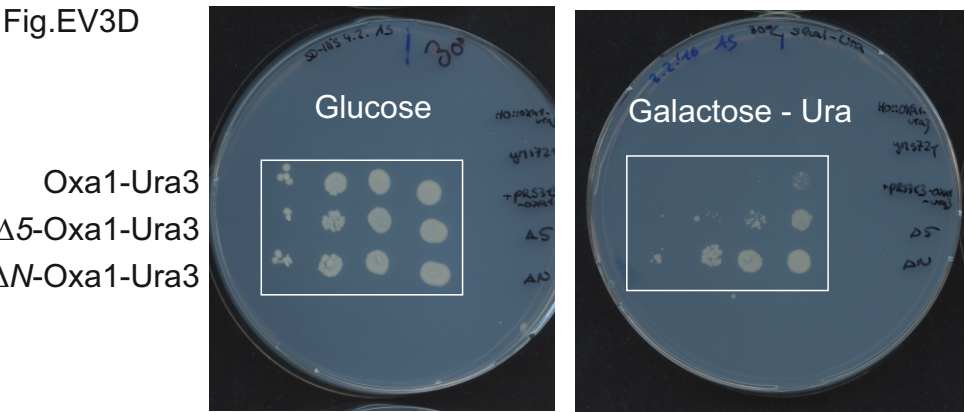

Supplement: Supplementary file 10 — Source Data for Expanded View [file EMBJ-40-e107913-s017.zip › EV_Figure_Source_Data/Source_Data_FigEV3.pdf]

Fig. EV4-A

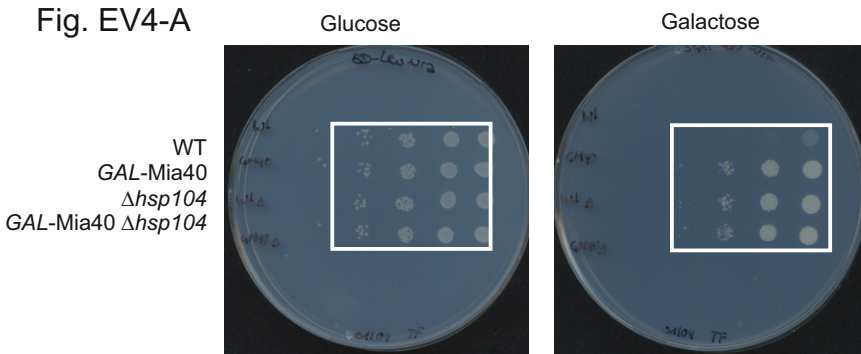

Fig. EV4-C

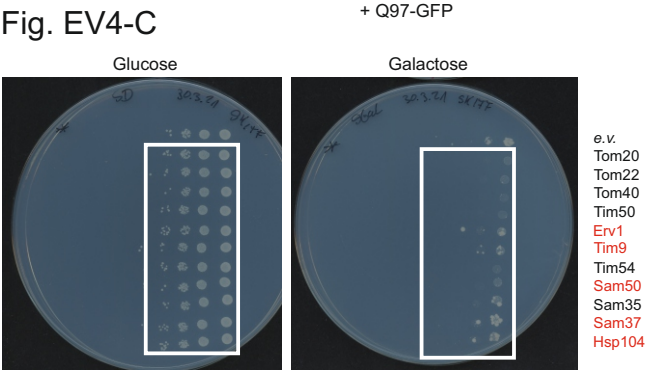

Fig.EV4D

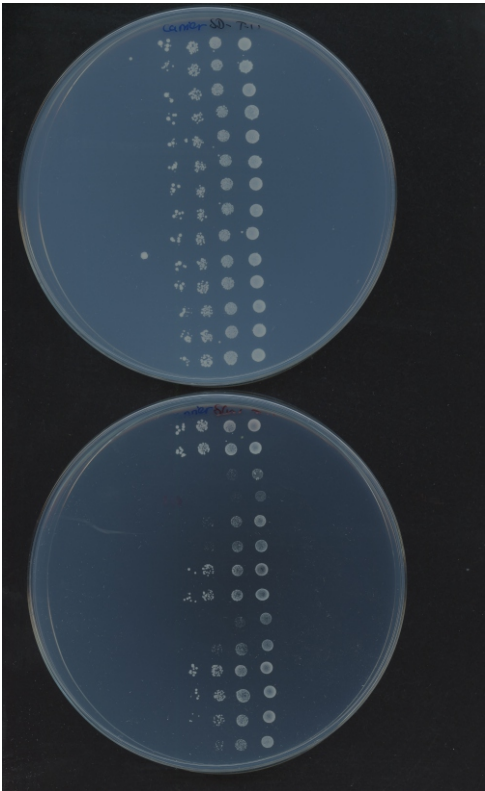

Fig.EV4E

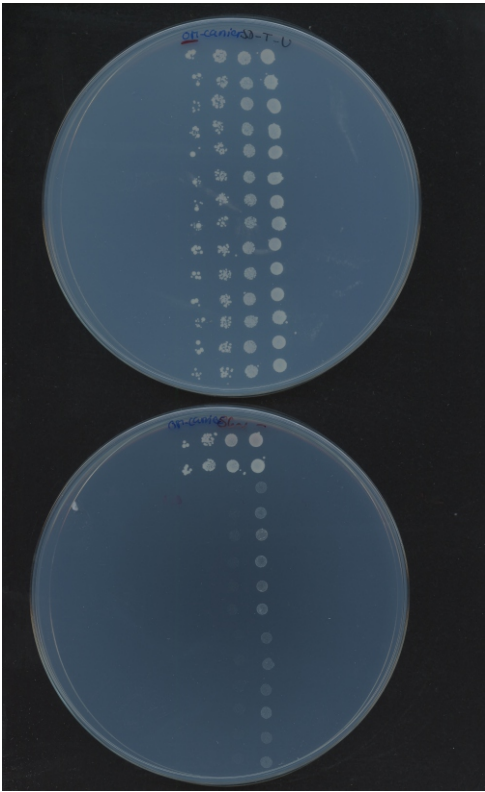

Fig.EV4F

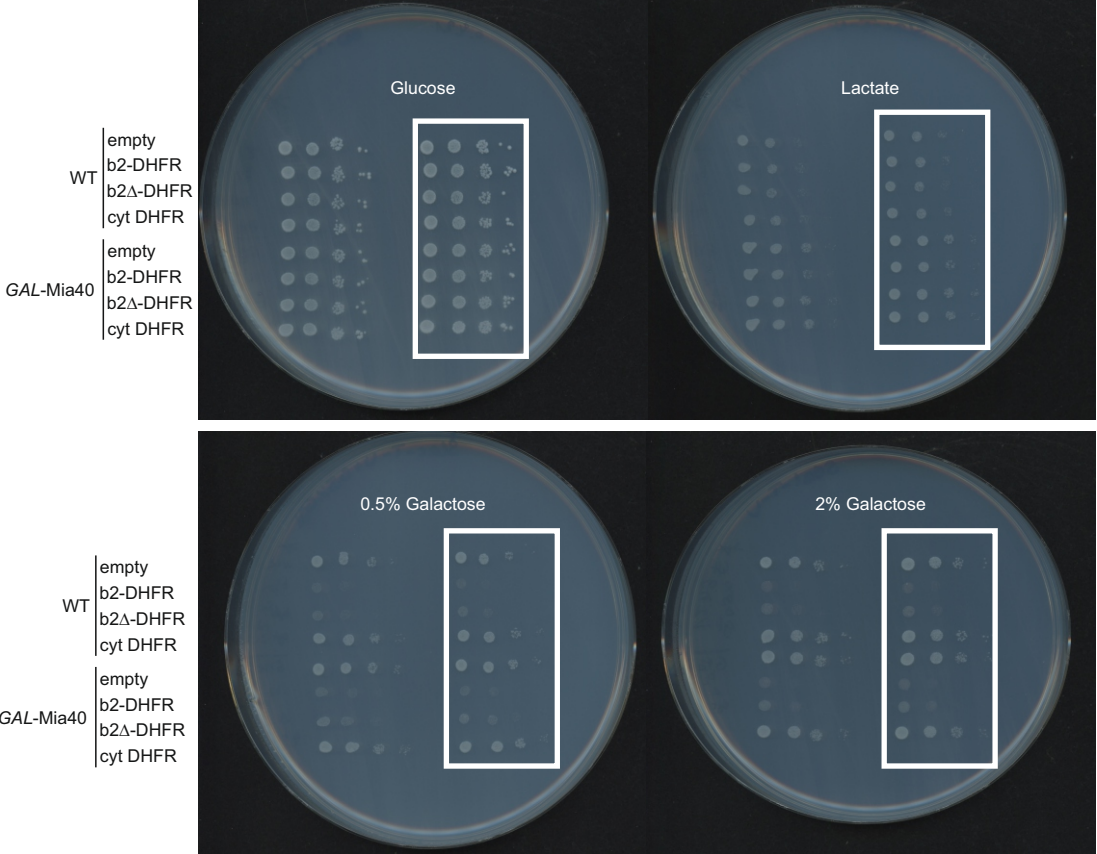

Supplement: Supplementary file 10 — Source Data for Expanded View [file EMBJ-40-e107913-s017.zip › EV_Figure_Source_Data/Source_Data_FigEV4.pdf]

Fig.EV5A

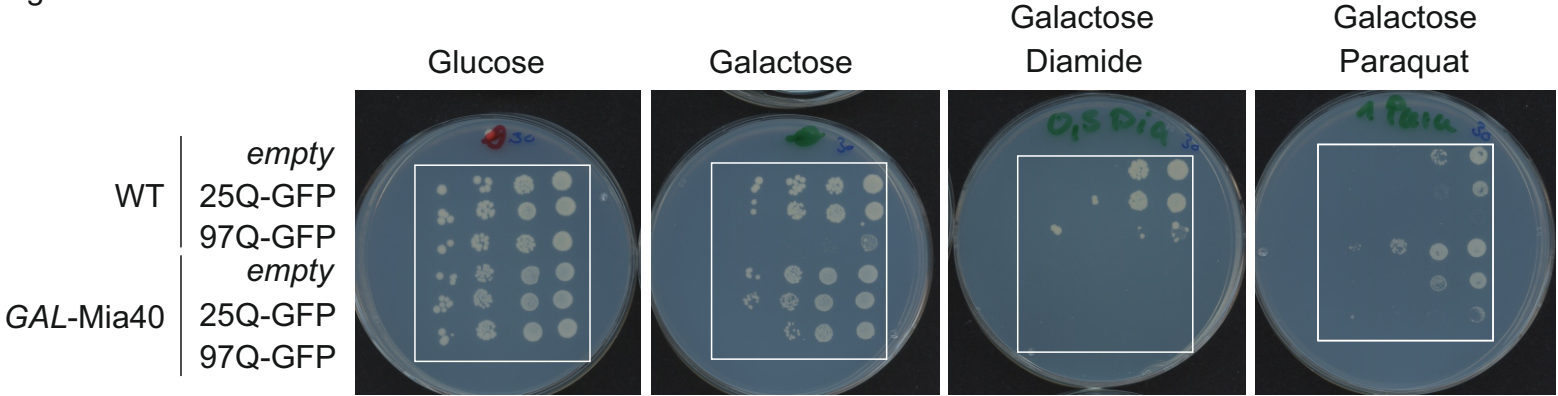

EV5B

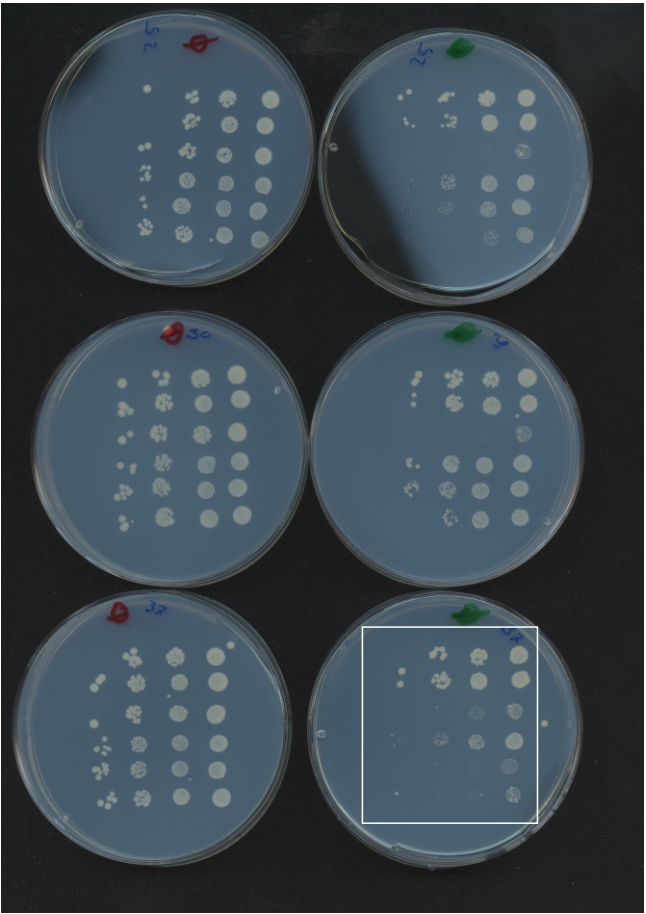

Supplement: Supplementary file 10 — Source Data for Expanded View [file EMBJ-40-e107913-s017.zip › EV_Figure_Source_Data/Source_Data_FigEV5.pdf]

Fig.1C

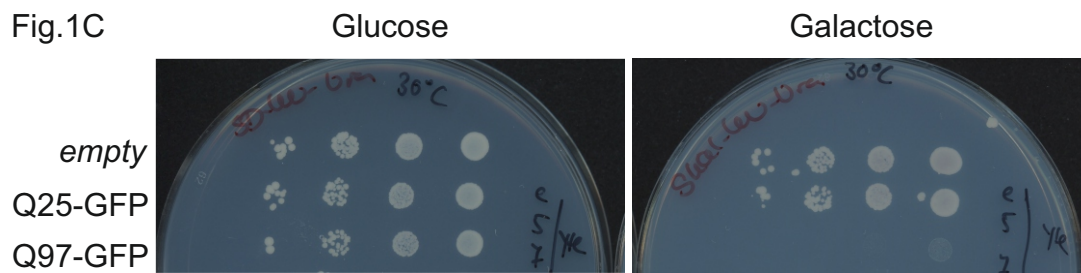

Fig.1E

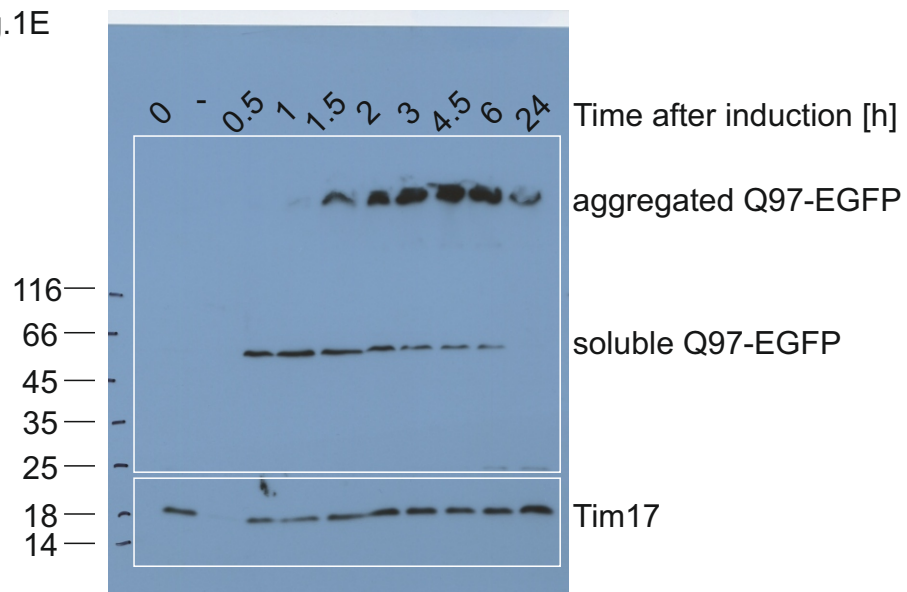

Supplement: Supplementary file 11 — Source Data for Figure 1 [file EMBJ-40-e107913-s006.pdf]

Fig.2D

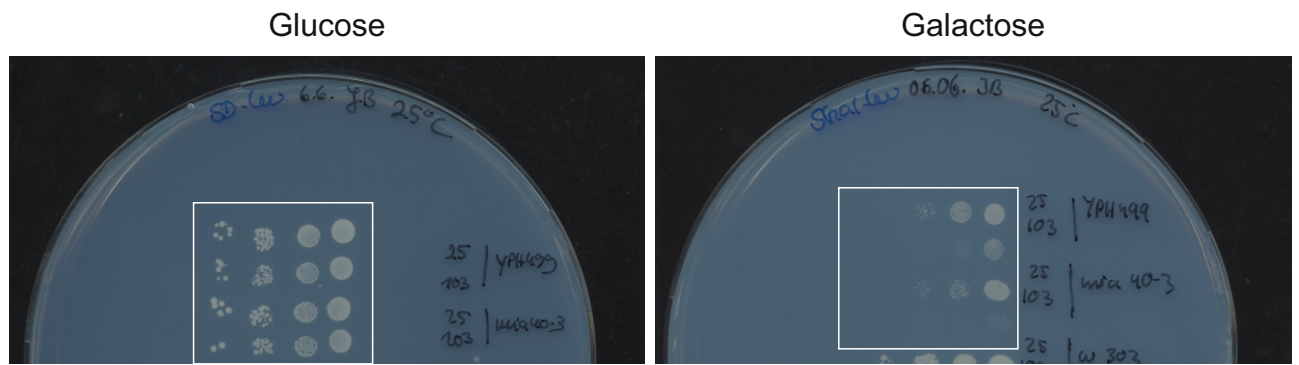

Fig.2E

t=0 h

t=4 h

t=6 h

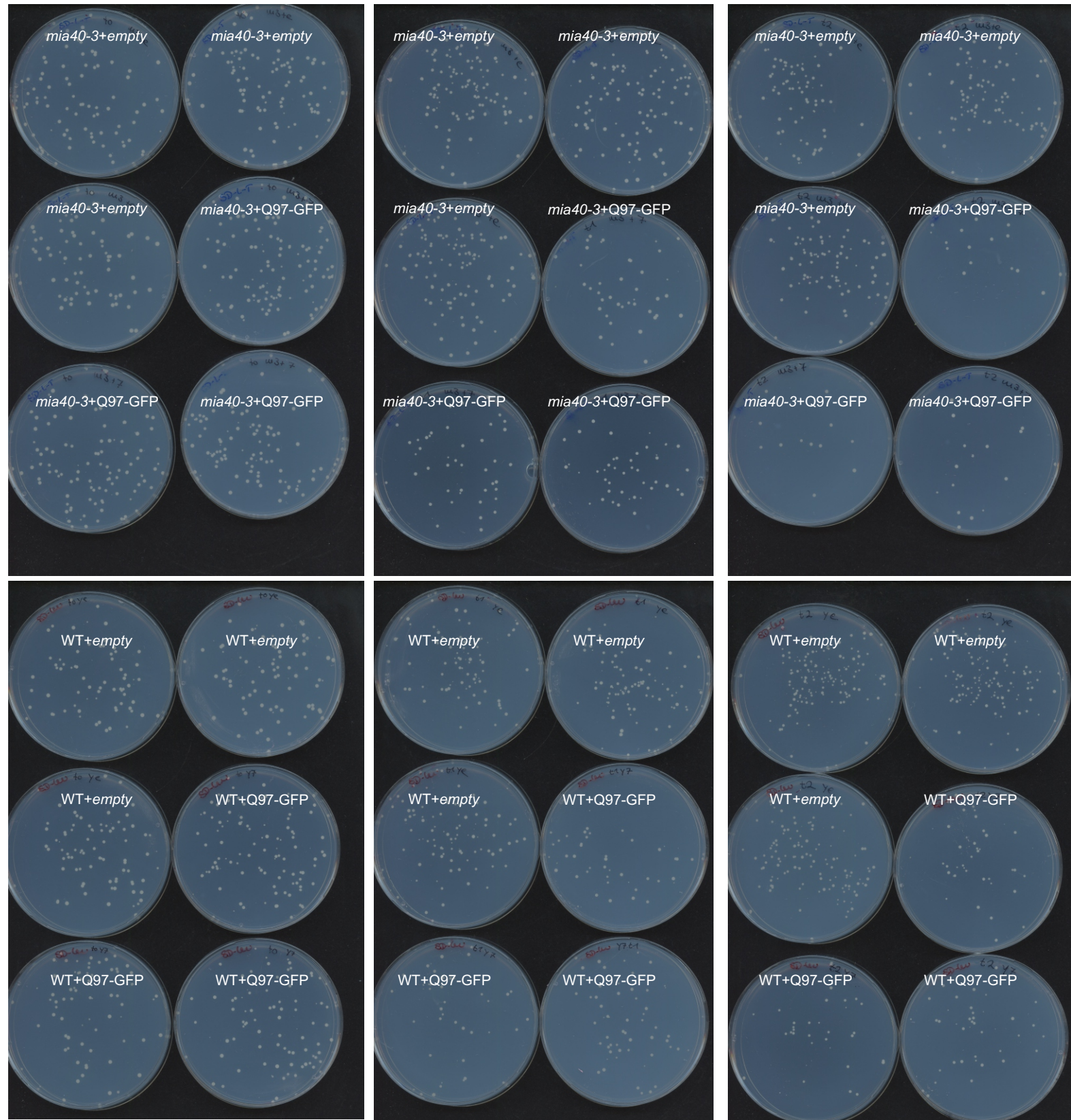

Supplement: Supplementary file 12 — Source Data for Figure 2 [file EMBJ-40-e107913-s008.pdf]

Fig.3A

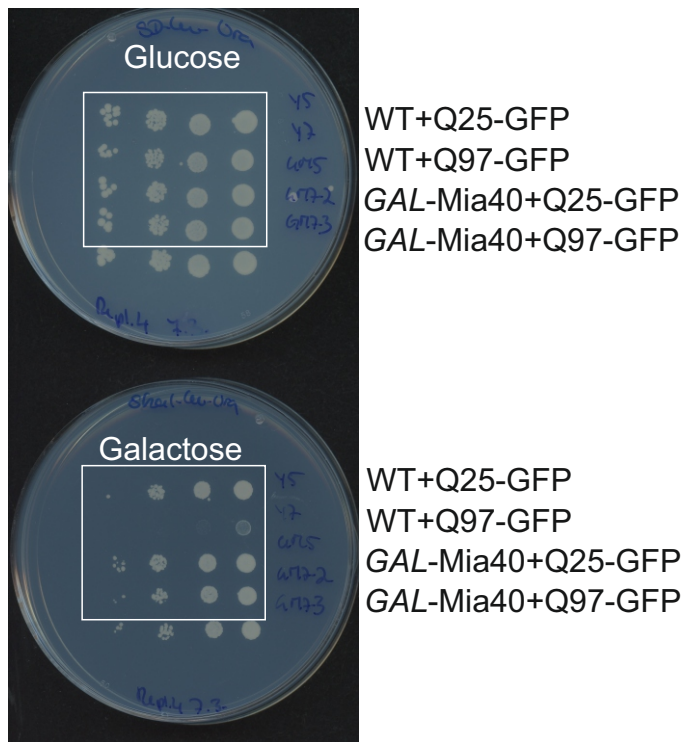

Fig.3B

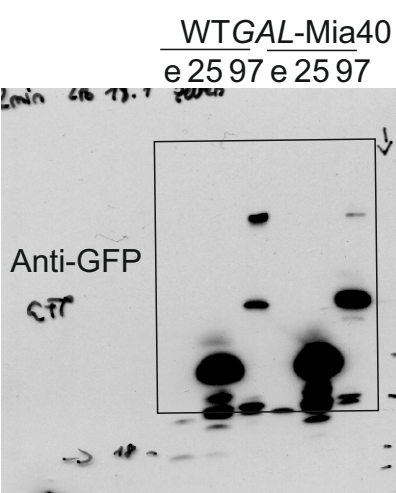

Supplement: Supplementary file 13 — Source Data for Figure 3 [file EMBJ-40-e107913-s004.pdf]

Fig.5D WT  $\Delta$ rnq1

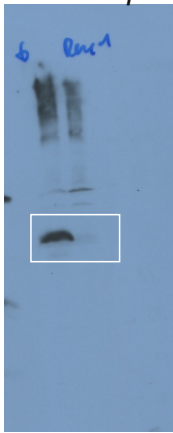

Fig.5E WT GAL-Mia40

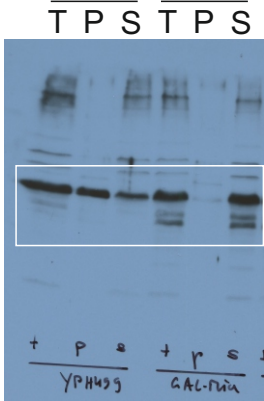

Fig.5F

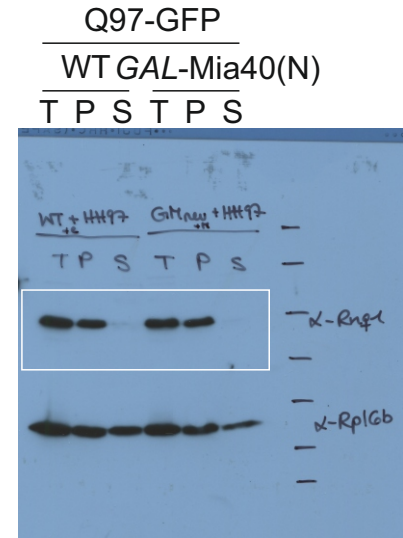

Fig.5G

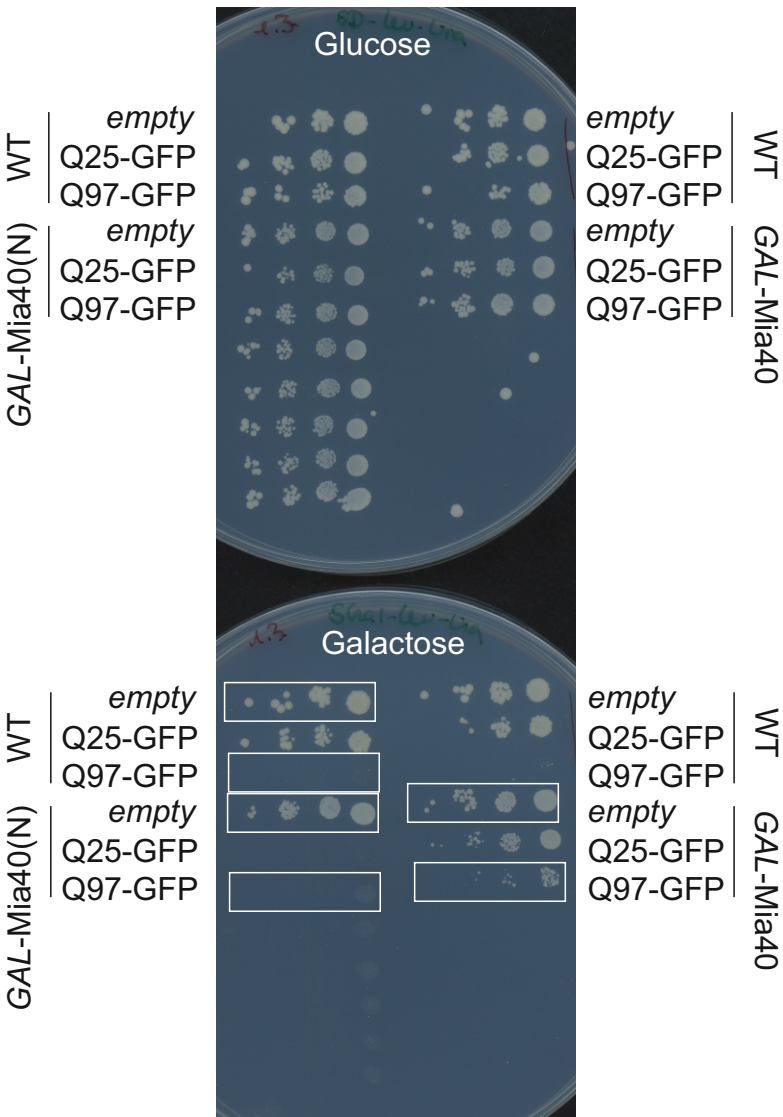

Fig.5I

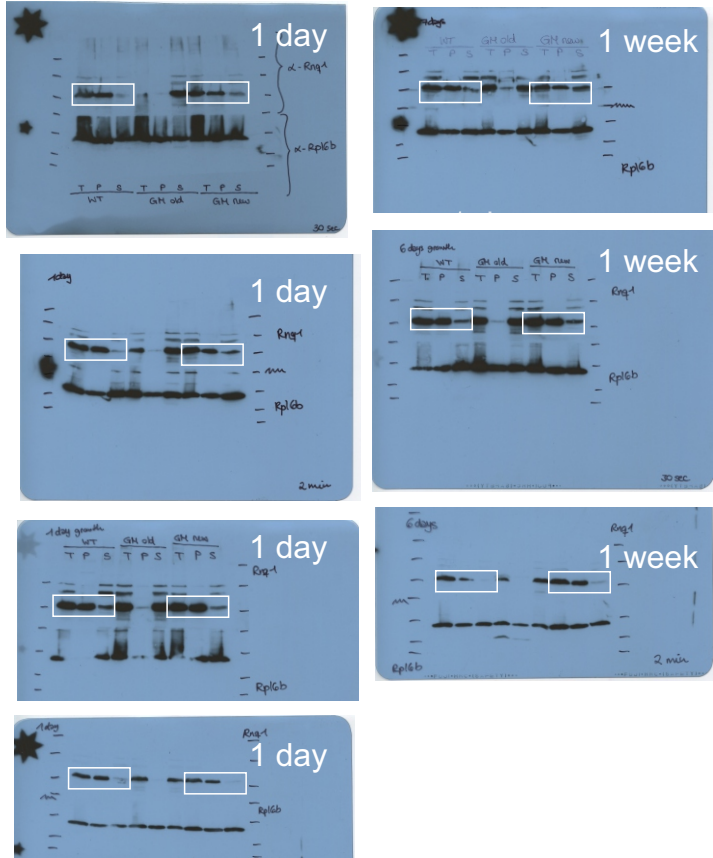

Supplement: Supplementary file 14 — Source Data for Figure 5 [file EMBJ-40-e107913-s015.pdf]

Fig. 7-B

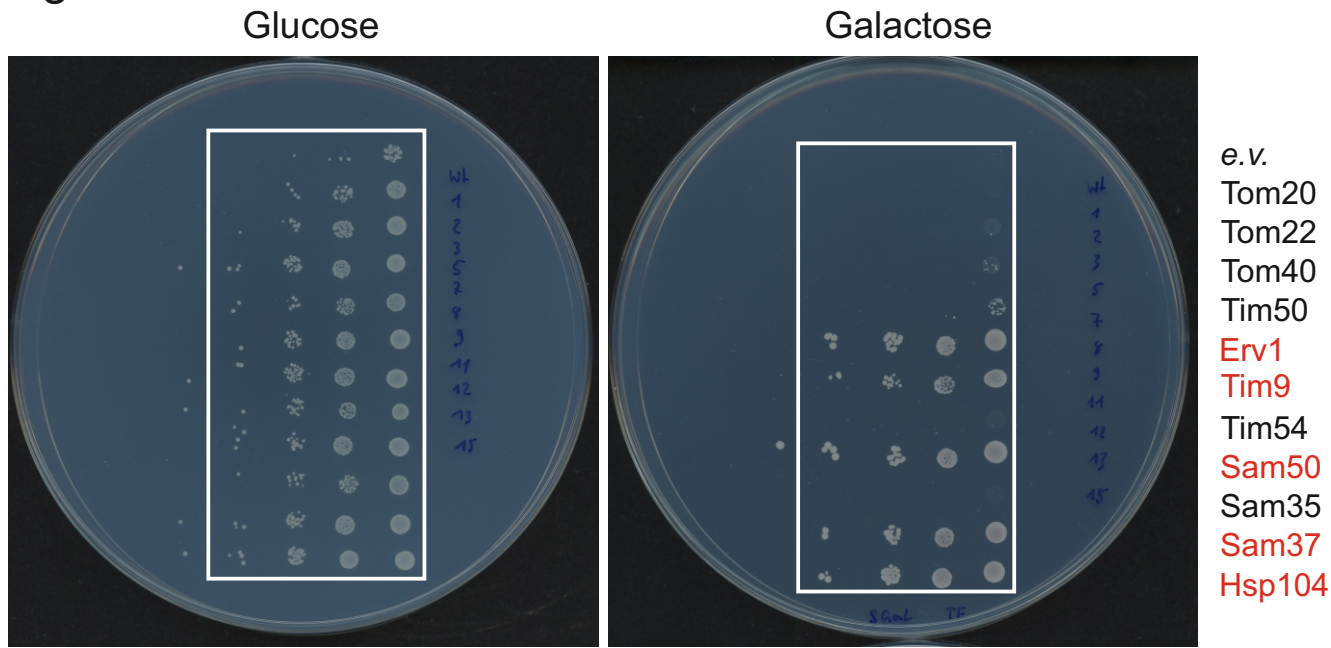

Supplement: Supplementary file 16 — Source Data for Figure 7 [file EMBJ-40-e107913-s011.pdf]

**A** same cell lysates were reloaded multiple times

Mia40

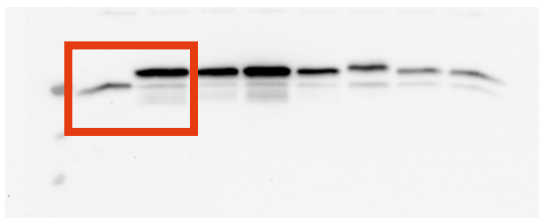

Strep

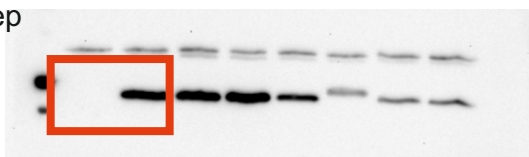

TCE

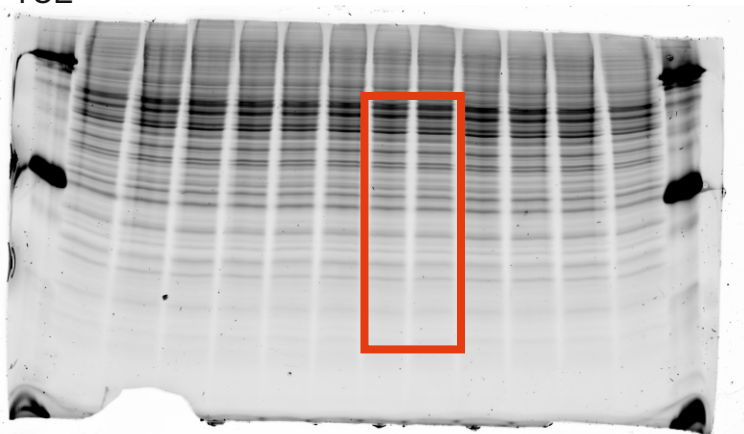

COX6B1

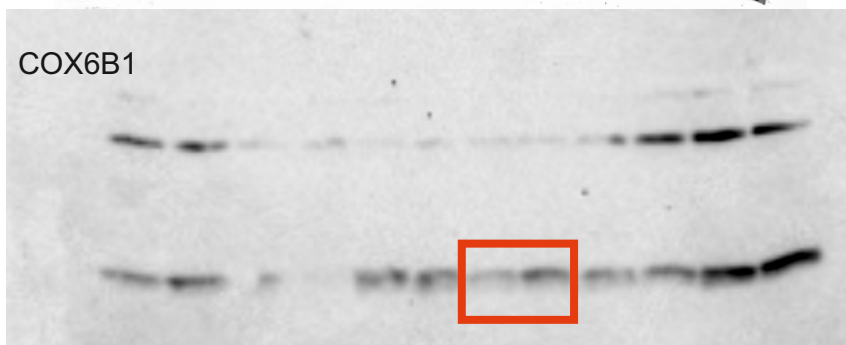

CMC1

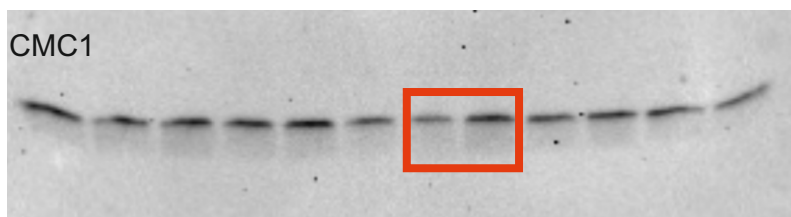

HSP70

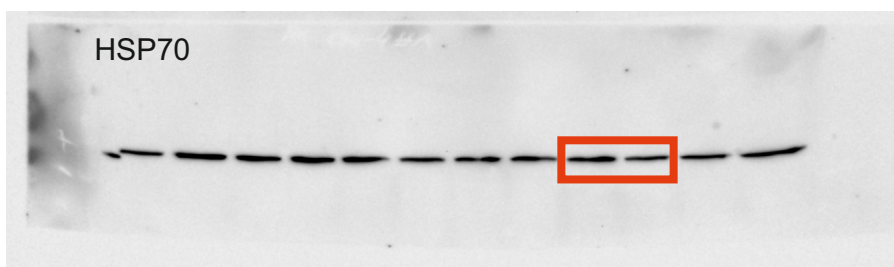

NDUFS5

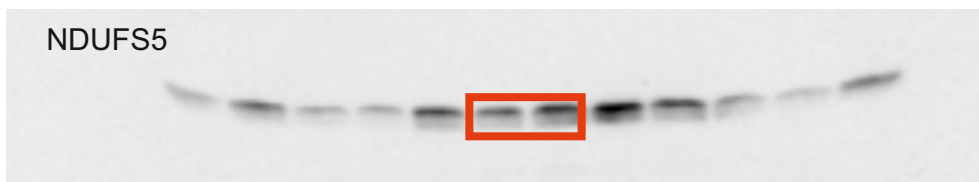

Supplement: Supplementary file 17 — Source Data for Figure 8 [file EMBJ-40-e107913-s002.pdf]
