# Supplementary material for: Increased levels of mitochondrial import factor Mia40 prevent the aggregation of polyQ proteins in the cytosol
Source: EMBO J. 2021 Jun 30;40(16):e107913. doi: 10.15252/embj.2021107913 (PMC8365258; doi:10.15252/embj.2021107913)

Fig.6A

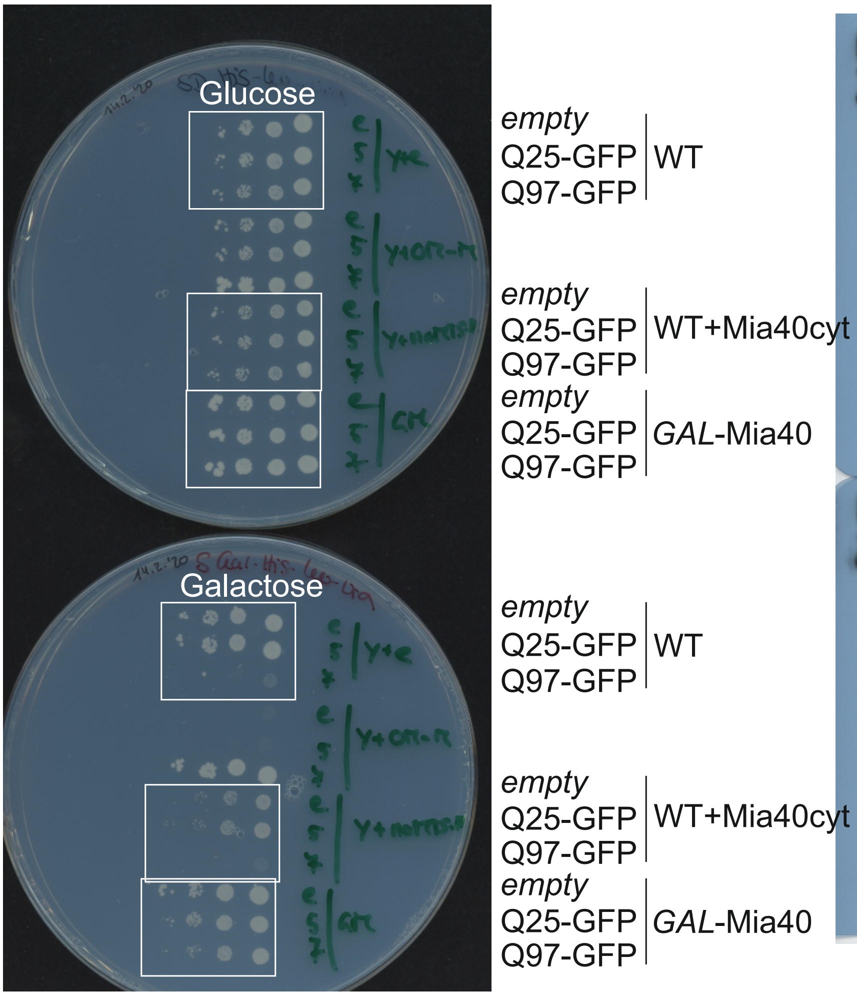

B

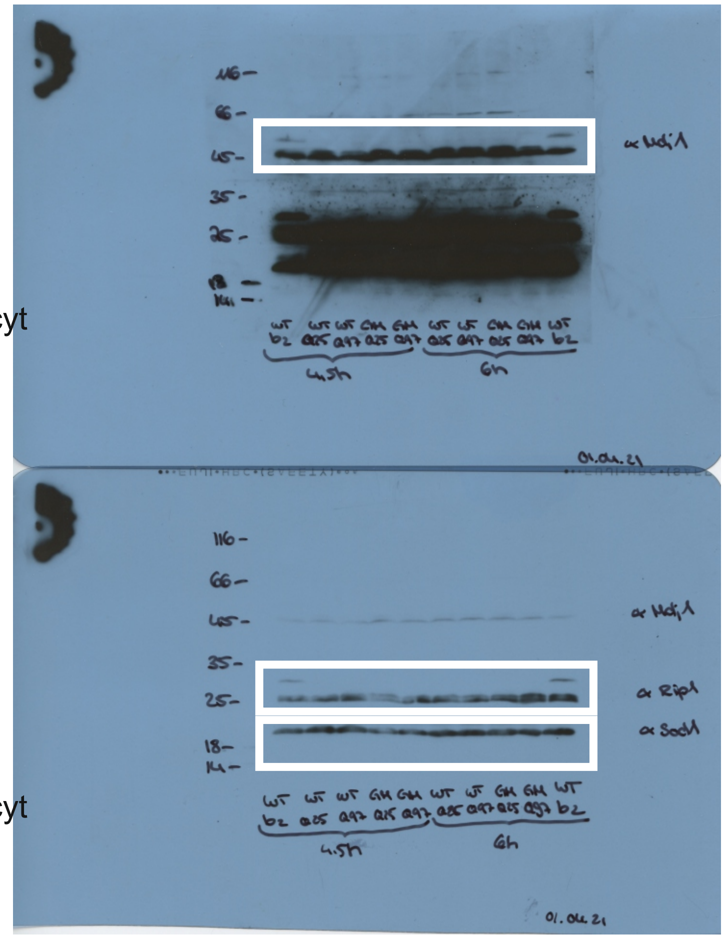

Fig.6C

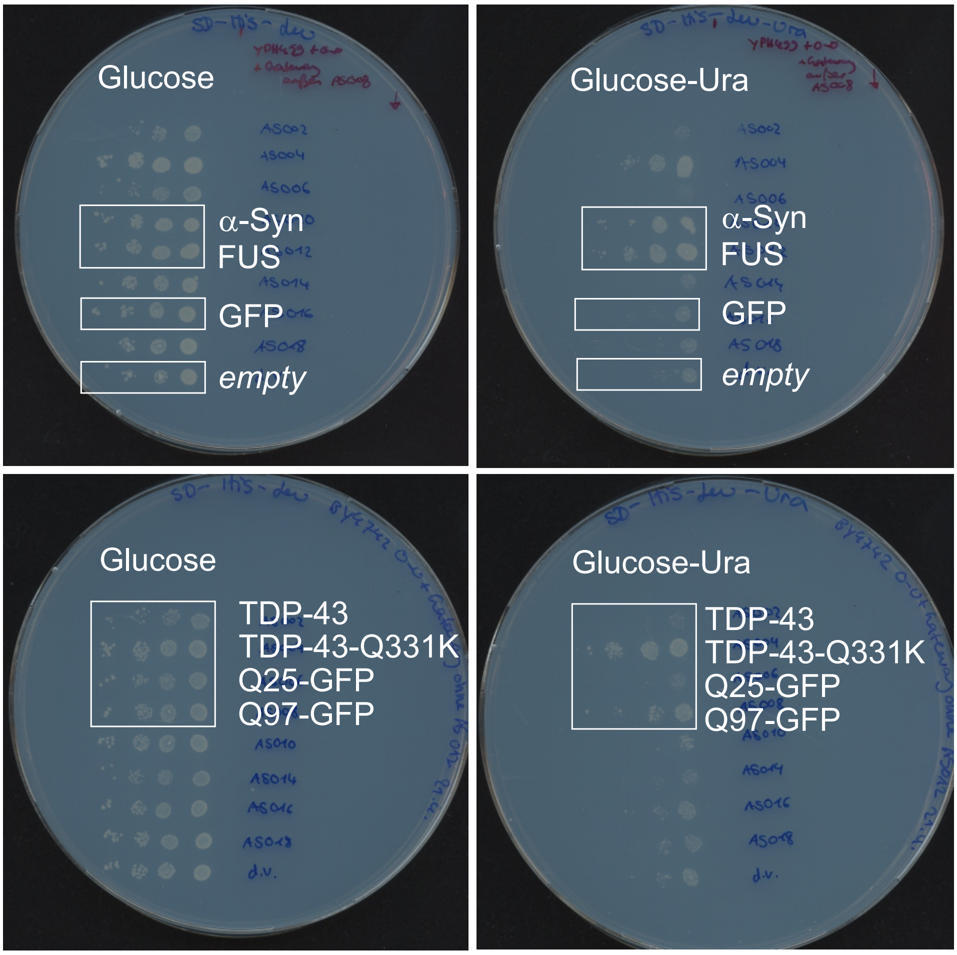

Supplement: Supplementary file 15 — Source Data for Figure 6 [file EMBJ-40-e107913-s016.pdf]
